# Extensive splicing changes in an ALS/FTD transgenic mouse model overexpressing cytoplasmic fused in sarcoma

**DOI:** 10.1038/s41598-020-61676-x

**Published:** 2020-03-17

**Authors:** Daisuke Ito, Ryota Taguchi, Maki Deguchi, Hideaki Ogasawara, Eiji Inoue

**Affiliations:** 1 Departments of Neurology, Keio University School of Medicine, 35 Shinanomachi, Shinjuku-ku, Tokyo 160-8582 Japan; 2 KAN Research Institute, Inc., 6-8-2 Minatojima-minamimachi, Chuo-ku, Kobe, Hyogo 650-0047 Japan

**Keywords:** Neuroscience, Diseases of the nervous system, Molecular neuroscience, Neurological disorders

## Abstract

Mutations in RNA-binding proteins (RBPs) such as TAR DNA-binding protein 43 (TDP-43) and fused in sarcoma (FUS) are associated with amyotrophic lateral sclerosis (ALS) and frontotemporal dementia (FTD). Recent evidence suggests that RNA dysregulation mediated by aberrant RBPs may play a critical role in neurodegeneration, but the underlying molecular mechanisms are largely unknown. In this study, we performed whole transcriptome profiling of various brain tissues of a transgenic (Tg) mouse model of ALS/FTD overexpressing the exogenous nuclear localization signal deletion mutant of human FUS (ΔNLS-FUS) to investigate changes associated with the early stages of ALS/FTD. Although there were not many differences in expression profiles between wild-type and Tg mice, we found that *Sema3g* was significantly upregulated in the frontal cortex and hippocampus of Tg mice. Interestingly, analysis of alternative splicing events identified widespread exons that were differentially regulated in Tg mice in a tissue-specific manner. Our study thus identified aberrant splicing regulation mediated by mutant FUS during the early stages of ALS/FTD. Targeting this aberrant splicing regulation represents a potential therapeutic strategy for ALS/FTD.

## Introduction

A landmark discovery in 2006 identified TAR DNA-binding protein 43 (TDP-43) accumulation as a pathological hallmark of both amyotrophic lateral sclerosis (ALS) and frontotemporal dementia (FTD), thus linking the disease mechanisms of ALS and FTD^1,2^. Further research identified seven ALS-related RNA-binding proteins (RBPs), FUS, TAF15, EWSR1, HNRNPA1, HNRNPA2B1, Matrin-3, and TIA1, that are characteristically deposited in the affected regions of the ALS/FTD brain^3^. These studies, along with others, strongly suggested that dysfunction in RNA processing mediated by aberrant RBPs is a key aspect of the neurodegeneration observed in ALS/FTD^3–7^.

We recently generated a transgenic (Tg) mouse model for ALS/FTD overexpressing the exogenous nuclear localization signal deletion mutant of human fused in sarcoma (ΔNLS-FUS), reflecting juvenile ALS^8,9^. The ΔNLS-FUS Tg mice show behavioral phenotypes such as cognitive deficits associated with FTD by nine weeks of age and show significant progressive motor impairment by five month of age, indicating that this Tg mouse line can be used as a model for ALS/FTD^8,9^. Histological and cytological studies of this Tg mouse model indicated that cytoplasmic FUS aggregates sequester mRNA and RNA transporters leading to synaptic and dendritic spine dysfunction resulting in FTD-like phenotypes even before the appearance of neuronal loss. These results suggest that a gain-of-function toxicity caused by cytoplasmic FUS aggregates lead to RNA dysregulation, thereby causing the behavioral and motor dysfunctions observed. Thus, a selective rescue of the surviving neurons may be an effective strategy for treating the motor deficits and even cognitive dysfunction observed in the early stages of ALS/FTD. Nevertheless, an important issue that needs to be addressed is which form of RNA dysregulation results in the neural dysfunction observed during the early stages of the disease.

Previously, RNA-seq transcriptome analysis of the ALS-linked mutant FUS Tg mice was performed, but they mainly concentrated on a single neuronal tissue isolated from older mice that show advanced phenotypes of ALS, such as neuronal loss^9,10^. The aim of this study is to investigate tissue-specific changes in RNA expression and alternative splicing events associated with the early stages of ALS/FTD using frontal cortex (FCx), hippocampus (Hp), and spinal cord (SC) tissues isolated from mutant FUS (ΔNLS-FUS) Tg mice at an age corresponding to the early symptoms of ALS/FTD. We performed this study to examine whether cytoplasmic accumulation of mutant FUS can induce aberrant splicing regulation or changes in RNA expression during the early stages of ALS/FTD in an effort to understand the mechanism of FUS proteinopathy.

## Results

### Sema3g is upregulated in the FCx and Hp tissues of Tg mice

To examine the underlying mechanism of FUS proteinopathy during the early stage of ALS and FTD, we first compared the transcriptome data generated from 6-month-old wild-type (Wt) and ΔNLS-FUS Tg mice, in which exogenously overexpressed mutant FUS (ΔNLS-FUS) was mislocalized in the cytoplasm (see Supplementary Fig. S1) and significant motor deficits appear^9^. We therefore performed whole transcriptome analysis of FCx, Hp, and SC tissues isolated from Wt and Tg mice (n = 4). On average, 23.27 ± 2.30 × 10^6^ reads from FCx tissue, 20.93 ± 2.99 × 10^6^ from Hp, and 23.88 ± 1.93 × 10^6^ from SC underwent quality control checks by FastQC and trimming by Trimmomatic. Hierarchical clustering and principal component analysis (PCA) showed a clear separation of each tissue, indicating good quality of the RNA samples and reads obtained (see Supplementary Fig. S2A). Surprisingly, no definite clustering pattern was detected between Wt and Tg mice, indicating that there were no major differences in transcriptome profiles between the two groups during early ALS/FTD (see Supplementary Fig. S2B–D).

Next, we examined the cell composition of each tissue sample by analyzing genes that are specifically expressed by neurons, motor neurons, astrocytes, oligodendrocytes, and microglia (see Supplementary Fig. S3 and Supplementary dataset 1: Cell composition). No significant changes in tissue-specific cell compositions were detected between Wt and Tg mice, indicating that gliosis and neuronal loss are not prominent in 6-month-old Tg mice.

**Figure 1:**
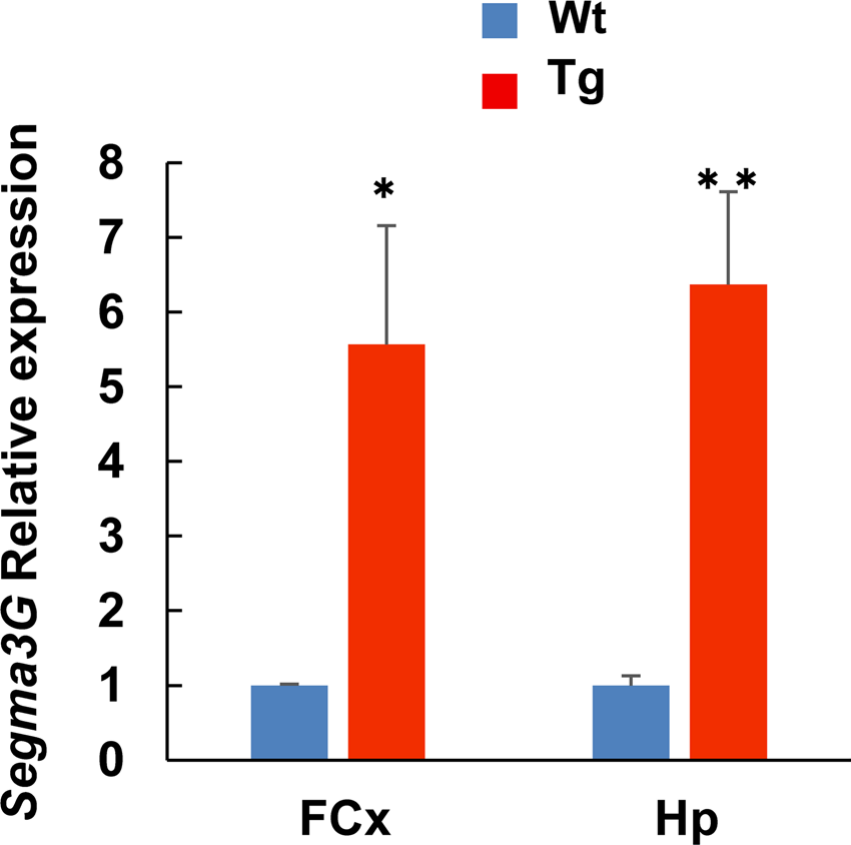
Relative expression of *Sema3g* in the brain tissues of wild-type (Wt) and ΔNLS-FUS transgenic (Tg) mice. qRT-PCR analysis of *Sema3g* expression was performed using RNA samples isolated from frontal cortex (FCx) and hippocampus (Hp) tissues from Wt and Tg mice (n = 4); expression of *Sema3g* was normalized to that of Gapdh. *P < 0.02, **P < 0.005.

We further probed for differentially expressed genes (DEG) that show a fold change (log2) ≥ 1 with adjusted p-values (p adj) < 0.05 between Wt and Tg mice in each tissue sample (see Supplementary dataset 2–4: Differentially expressed genes_FCx, Hp or SC). As shown in Table 1, only a few genes showed significant differences in expression levels; in Tg mice, *Sema3g* in FCx and Hp tissues was upregulated (Tg/Wt; 2.60 and 3.64 fold, respectively) and *A830039N20Rik* in FCx tissue was downregulated, while four genes in SC tissue were upregulated and another four (*Hbb-b2, Hba-a2, Hba-a1*, and *Hbb-b1*) downregulated (see Supplementary Table S1). We further analyzed *Sema3g, Hbb-b2, Hba-a2*, and *Alas2* expression levels in four Tg and Wt mice by qRT-PCR and confirmed that *Sema3g* expression is significantly increased in FCx and Hp tissues of Tg mice (Tg/Wt; 5.56 and 6.37 fold, respectively; Fig. 1), but the decrease in expression levels observed for *Hbb-b2, Hba-a2*, and *Alas2* in SC tissues was not significant (p = 0.105, p = 0.113, and p = 0.321, respectively).

**Table 1:** List of differentially expressed genes (padj < 0.05, |log2FC | ≧ 1) in FCx and Hp tissues of ∆NLF-FUS Tg mice.

Tissue	Gene_name	log2 Fold Change	adjusted p-value	Wt. ave. ± S.D. (FPKM)	Tg ave. ± S.D. (FPKM)	Description	KEGG Pathway
FCx	Sema3g	1.41	7.55E-23	2.00 ± 0.17	5.19 ± 0.35	sema domain, immunoglobulin domain (Ig), short basic domain, secreted, (semaphorin)	Axon guidance
	A830039N20Rik	−1.23	0.01	37.81 ± 10.67	15.69 ± 5.98		
Hp	Sema3g	1.85	6.80E-15	1.71 ± 0.24	6.22 ± 0.71	sema domain, immunoglobulin domain (Ig), short basic domain, secreted, (semaphorin)	Axon guidance

### Tg mice exhibit widespread differential alternative splicing events

**Figure 2:**
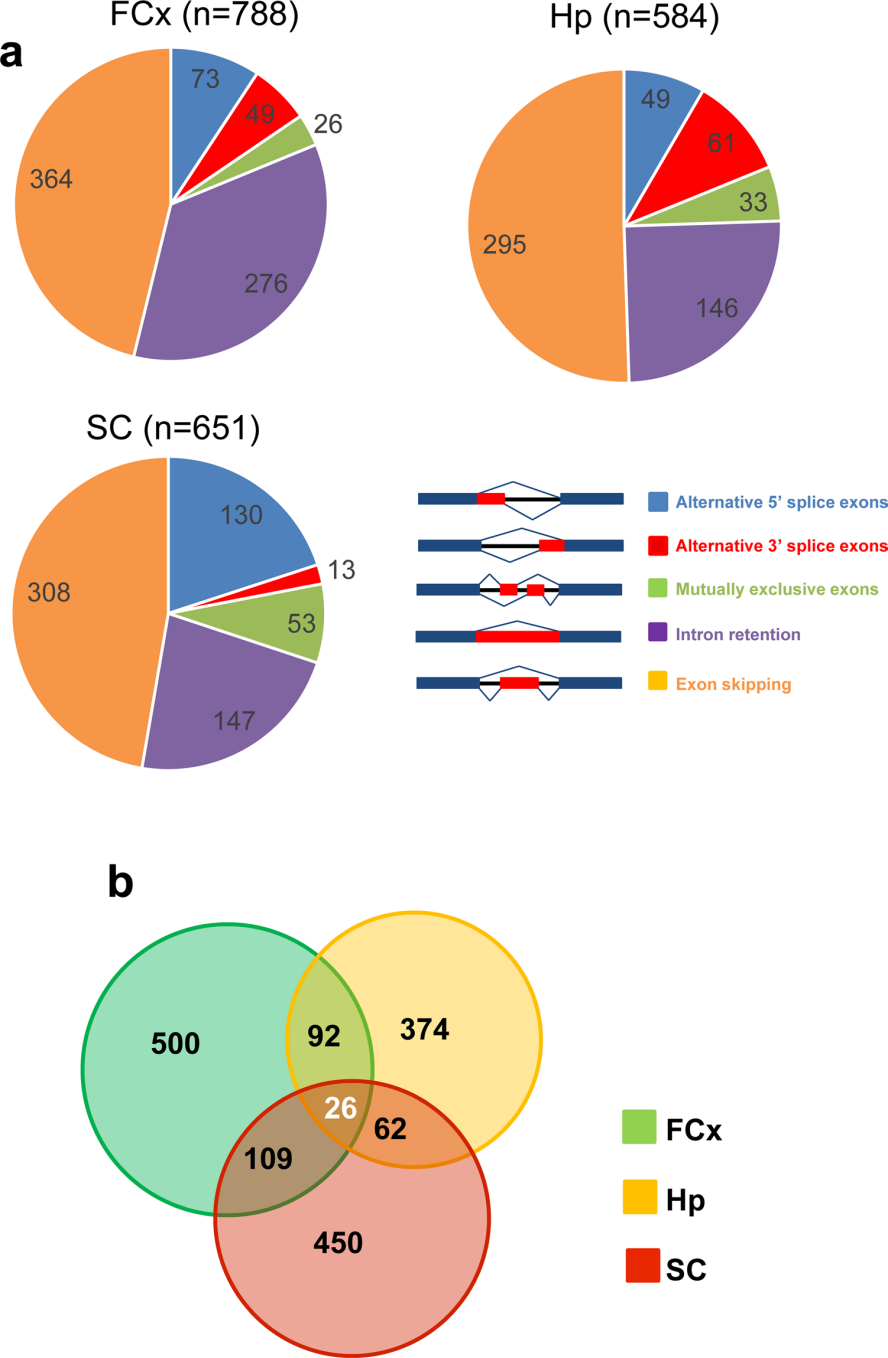
Differential alternative splicing events in the ∆NLS-FUS transgenic (Tg) mice. (**a**) Pie charts displaying the total number of alternative splicing events identified as differentially regulated in the frontal cortex (FCx), hippocampus (Hp), and spinal cord (SC) tissues of Tg mice *vs*. Wt mice; colors represent alternative splicing subtypes. (**b**) Venn diagram showing the overlap between differential alternative splicing events across the three tissues studied.

To further explore the underlying mechanism of FUS proteinopathy, we investigated the differential alternative splicing events between Wt and Tg mice using the transcriptome data generated from the tissue samples. Analysis of alternative splicing events revealed that 788, 584, and 651 exons in the FCx, Hp, and SC tissue samples, respectively, were differentially regulated in Tg mice; we used a statistical threshold that captured as many significant changes as possible (FDR < 0.05; Fig. 2a, also see Supplementary dataset 5–7; Splicing changes_FCx, Hp, or SC). When we categorized the observed changes among the alternative splicing subtypes, namely alternative 5′ and 3′ splice sites, mutually exclusive exons, intron retention, and exon skipping, we found that the most frequent subtype was exon skipping across all tissues (Fig. 2a). Notably, 26 genes showed differential alternative splicing in all tissues (Fig. 2b), two of which (Slc35f3 and Zscan2) belonged to the same alternative splicing subtype and direction. We further investigated the pathways associated with these genes using Ingenuity Pathway Analysis (IPA; p < 0.05) and identified diverse pathways for each tissue sample with only three pathways (apelin cardiomyocyte signaling pathway, calcium signaling, Huntington’s disease signaling) showing an overlap (Supplementary dataset 8: IPA_splicing.xlsx); this indicates that under mutant FUS (ΔNLS-FUS) overexpression, aberrant splicing regulation varies and is tissue-dependent. To explore our data further, we narrowed our study to 73 alternative splicing events in 72 genes under more stringent conditions (p < 0.001 and |IncLevelDifference| > 0.5) and identified 18 proteins with varying functions (RNA dysregulation, protein quality control, neurotransmitter, vesicular transport, microtubule regulation) that are possibly associated with the neurodegenerative pathology of ALS/FTD (Table 2).

**Table 2:** List of proteins encoded by the alternative mRNA changed in ΔNLF-FUS mice. Genes were determined using referred functions linked neurodegeneration.

Tissue	splicing category	Gene_name	longES	longEE	shortES	shortEE	flankingES	flankingEE	Pvalue	IncLevelDifference	Description
FCx	A5SS	Prpf40b	99125671	99125859	99125671	99125704	99134471	99134615	######	0.53	Pre-mRNA splicing.
	SE	Lypd6b	49695607	49695663	49643250	49643293	49786444	49786504	######	0.742	Modulator of nicotinic acetylcholine receptors. Apositive regulator of Wnt/β-catenin signaling.
		Nrg2	36191989	36192066	36183959	36184062	36197884	36198005	######	−0.59	Signaling receptor binding and growth factor activity. Candidate for Charcot-Marie-Tooth disease.
		Kif9	110380502	110380745	110379497	110379587	110385564	110385662	######	−0.587	Microtubule cytoskeleton. Golgi-to-ER retrograde transport.
Hp	A5SS	Otof	30682475	30682602	30682475	30682546	30682896	30683059	######	0.505	Calcium ion sensor involved in synaptic vesicle-plasma membrane fusion and in the control of neurotransmitter release.
	SE	Odz3	49731611	49731890	49502361	49502599	49759763	49760044	######	−0.609	Pre- and postsynaptic neurons in the hippocampus to control the assembly of a precise topographic projection.
		Cux2	122493778	122493853	122374813	122374924	122500024	122500111	######	−0.516	Transcription factor involved in neural specification.
		Cops4	100953029	100953096	100947755	100947954	100956992	100957072	######	0.518	Regulator of the ubiquitin (Ubl) conjugation pathway
		Lyrm7	54662080	54662898	54653314	54654704	54663846	54663917	######	−0.62	Chaperone, binding to this subunit within the mitochondrial matrix
SC	A5SS	Rnf180	106042331	106042430	106042331	106042427	106060984	106061119	######	0.672	E3 ubiquitin-protein ligase which promotes polyubiquitination and degradation.
		Ahdc1	132568104	132568202	132568107	132568202	132567420	132567438	######	0.657	Diseases associated with Xia-Gibbs Syndrome and Sleep Apnea.
		Grin2c	115122047	115122558	115122047	115122461	115128303	115128557	######	−0.589	Component of NMDA receptor complexes.
	SE	Stmn1	134025935	134026090	134024320	134024344	134028718	134028840	######	−0.725	Regulation of the microtubule filament system.
		Golgb1	36885096	36885274	36875225	36875349	36886216	36886314	######	−0.584	Forming intercisternal cross-bridges of the Golgi complex.
		Kalrn	34049915	34050008	34039964	34040095	34053645	34053750	######	−0.546	Promotes the exchange of GDP by GTP, inducing signaling mechanisms that regulate neuronal shape, growth, and plasticity.
		Whrn	63090119	63090152	63079121	63079656	63092883	63093036	0.0002	−0.523	Organization and stabilization of sterocilia elongation and actin cystoskeletal assembly.
		Cox19	139814693	139814796	139814093	139814329	139815135	139815339	######	0.5	Assembly of mitochondrial cytochrome c oxidase.
		Dda1	73996093	73996145	73993097	73993224	73998386	73999997	######	0.502	Ubiquitination and proteasomal degradation of target proteins.

ES, exon start; EE, exon end; A5SS, alternative 5′ splice exons; A3SS, alternative 3′ splice exons; SE, skipping exon.

IncLevelDifference = average (inclusion level for Wt replicates calculated from normalized counts) – average (inclusion level for Tg).

**Figure 3:**
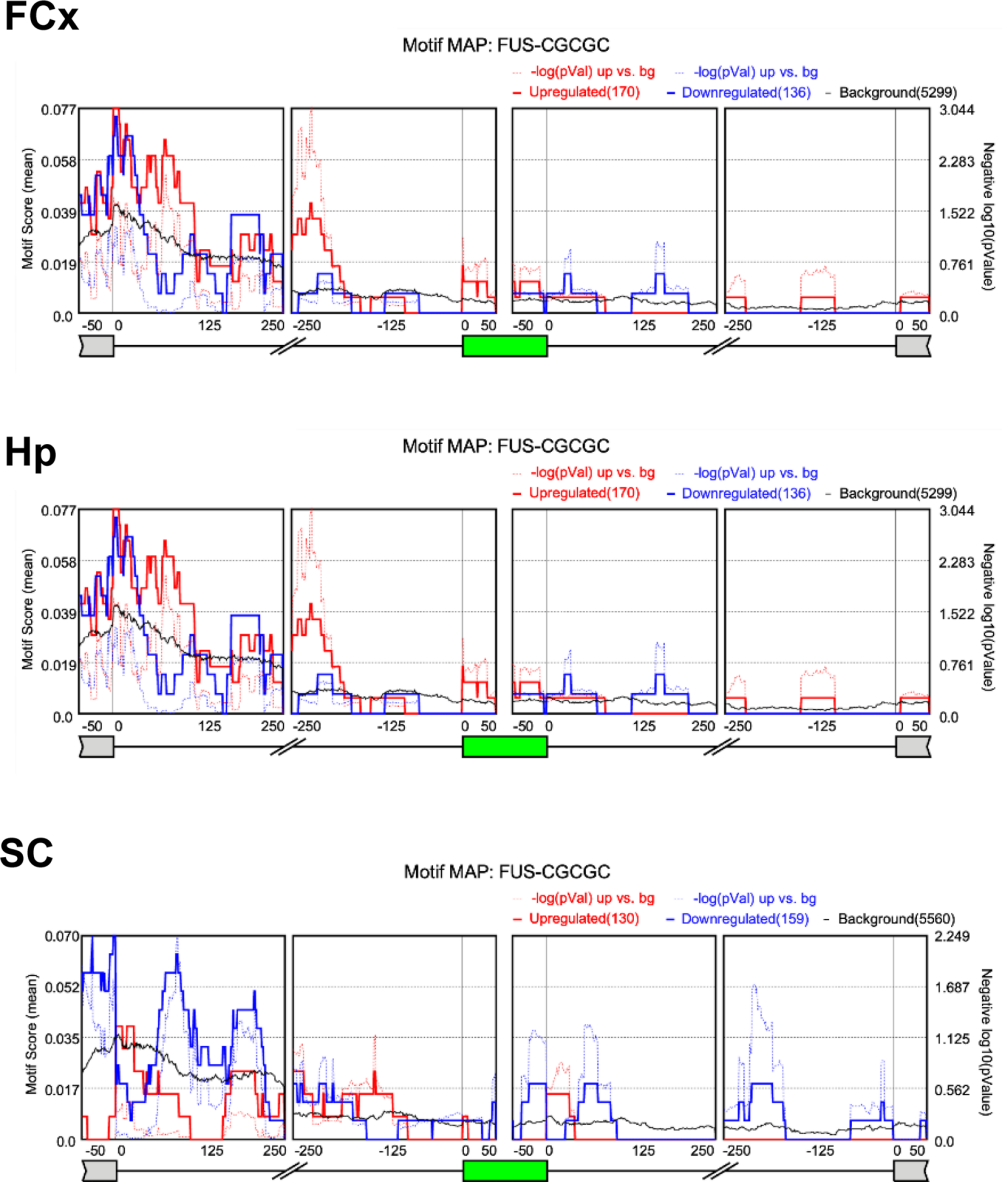
RNA-maps depicting the spatial distribution of FUS motifs in the vicinity of differential alternative splicing events, belonging to the exon-skipping subtype, identified in the Tg mice. The red line represents the enriched motif for enhanced exons, the blue line represents the enriched motif for silenced exons, and the black line represents the motif density for background (non-regulated) exons in the Tg mice. Solid lines represent the peak quality Motif score (peak height) as scaled on the left. Dotted lines represent the negative log10 (P-value) as scaled on the right. The green box indicates the cassette exon.

To identify the role of RBPs in the regulation of the differential alternative splicing events identified, we analyzed known RBP motifs and binding sites around the differentially regulated alternatively spliced exons belonging to the exon-skipping subtype using rMAPS, which systematically generates RNA-maps depicting the spatial distribution of RBP motifs in the vicinity of these alternatively spliced exons (see Supplementary dataset 9–14; rMAPS. up or down_FCx, Hp or SC). The RNA-map generated for FUS, depicting the spatial distribution of the FUS motif around differential exon skipping events, showed FUS motif enrichment downstream (~50–125 bp) of the silenced and enhanced exons in FCx and Hp tissues, whereas enrichment of silenced exons was more dominant in SC tissue (Fig. 3). Notably, our analysis further revealed that other RBPs including those known to be associated with ALS and/or stress granule formation, i.e., FMR1, hnRNPA1, hnRNPA2B1, and Matrin-3, also showed enrichment around differentially regulated alternatively spliced exons in the Tg mice (Supplementary Fig. 4–6. Thus, our motif analysis suggests a potential role not just for FUS but for various RBPs in the aberrant splicing regulation observed resulting from mutant FUS expression in the Tg mice.

## Discussion

Several studies have demonstrated that many RBPs with known mutations associated with ALS/FTD accumulate in the cytoplasm of the affected regions of the central nervous system and possibly contribute to the RNA dysregulation implicated in ALS/FTD pathogenesis^3–6,11^. Our previous study showed that the cytoplasmic mutant FUS aggregates observed in the ΔNLS-FUS Tg mice sequester mRNA and RNA transporters, leading to a disturbance in RNA/protein homeostasis in the dendrites, even before neuronal loss occurs^8,9^. Our current study provides valuable insights regarding the RNA dysregulation associated with the early stages of ALS/FTD before neuronal loss.

Whole transcriptome analysis identified *Sema3g* as significantly upregulated in FCx and Hp tissues isolated from the Tg mice. This is in agreement with the brain transcriptome profiles reported for sporadic ALS (NCBI Gene Expression Omnibus; accession number: GSE67196), which shows an increase in the expression of *Sema3g* in FCx^12^. Furthermore, transcriptome analysis performed during our previous study on the whole cerebrum of 1-year-old ΔNLS-FUS Tg mice also showed a 10-fold upregulation in *Sema3g* (Wt, 0.22 FPKM; Tg mice, 2.31 FPKM)^9^; RNaseL, Kcnh7, Atp6ap1, Npl, and Hspa5 showed similar changes in expression levels to that of the previous study, but the differences were not significant. Interestingly, *Sema3g* was recently found involved in the regulation of synapse density and plasticity of hippocampal neurons through its neuronal neuropilin 2/plexin-A4 holoreceptor^13^. *Sema3g*-deficient mice are reported to show disturbances in hippocampal-dependent memory^13^. Moreover, our previous study on ΔNLS-FUS Tg mice showed a decrease in dendritic spine and synaptic density in the Hp, leading to an impairment of hippocampal memory retrieval^8^. These studies indicate a critical role for *Sema3g* in hippocampal synapse regulation in neurodegenerative diseases, and our current study suggests a possible role for *Sema3g* in the early stages of ALS/FTD. Therefore, we propose *Sema3g* as a potential biomarker for the early stages of ALS/FTD and a potential therapeutic target in various cognitive disorders.

Our study also identified a large number of differentially regulated, alternatively spliced exons in the 6-month-old ∆NLS-FUS Tg mice, which corresponds to the early stages of ALS/FTD. Pathway analysis indicated that these exons were associated with different pathways in a tissue-specific manner. Previous studies on FUS-knockout mice demonstrated a major role of FUS in regulating specific subtypes of alternatively spliced exons^14^; 77% of cassette exons increased their inclusion in the knockout brain^14^; however, the rate was only 54.1% (551/1020 in all tissues, 47.5% in FCx, 57.5% in Hp, and 57.3% in SC) in our study. The reported 20 splicing changes in the FUS-knockout mouse brain^14^ did not exhibit similar changes in our Tg mice. The only exception, showing the same direction, was Dtna in SC tissue, which was an increased inclusion of a cassette exon [alternative exon (mm9), location chromosome 18: 23655979–23656204, IncLevelDifference = −0.101, p = 0.0006, and FRD = 0.0481]. This may be because the aberrant splicing regulation mediated by the FUS mutant ΔNLS-FUS is not similar to the FUS loss-of-function mutant. It is of note however, that the aberrant splicing mediated by the FUS mutant leads to the neurological deficits observed at the early stages of ALS/FTD.

The previous study as well as the current experiments showed that the mutant FUS protein is strictly localized in the cytoplasm, whereas splicing events occur in the nucleus (see Supplementary Fig. S1) and no evident changes in expression level or nuclear localization of endogenous FUS^8,9^ were observed, suggesting that the aberrant splicing observed in ΔNLS-FUS Tg mice may be due to an indirect effect, and that mutant FUS may be interfering with other factors involved in splicing regulation in a toxic gain-of-function manner. Furthermore, recent evidence showed that the mutant FUS aggregates sequester mRNA and associated proteins including spliceosomes in the cytoplasm^8,9,15–17^. Therefore, we investigated the role of other RBPs in regulating the differential alternative splicing events identified. As rMAPS analysis revealed various RBPs enriched around the cassette exons of differentially regulated exon skipping events, we propose that the mutant FUS possibly interferes with other RBPs, perturbing the splicing machinery and resulting in aberrant splicing regulation and RNA dysregulation.

There are several limitations associated with the study. First, we cannot exclude the possibility that the induced expression of mutant FUS may itself affect neuronal functions. However, in our previous study, we confirmed that the expression level of exogenous FUS was ~80% of that of endogenous FUS and that nuclear localization of endogenous mouse FUS was not affected by mutant FUS expression^9^. Nonetheless, Tg mice overexpressing even wild-type FUS were reported to show pathologies associated with motor neuron degeneration reminiscent of ALS^18,19^. Recent genomic analysis identified four mutations in the 3ʹ untranslated region of FUS—which increases the expression of native FUS—that are associated with sporadic and familial ALS^20,21^. Therefore, it was suggested that increased expression of even wild-type FUS can affect RNA regulation and cause ALS-associated symptoms^3,20,21^. Furthermore, in previous studies, other RNA-binding proteins (FMR1, staufen, and G3bp) and various mRNA were trapped in the cytoplasmic ΔNLS-FUS aggregates, suggesting that these aggregates directly lead to RNA dysregulation without interference from endogenous FUS^8,9^. Although we cannot rule out that cytoplasmic aggregates of ΔNLS-FUS sequester undetectable levels of endogenous FUS, our data indicate that mislocalized mutant FUS results in aberrant splicing in a toxic gain-of-function manner.

Second, our transcriptome analysis was limited to 6-month-old Tg mice that developed FTD-like behavioral deficits and had decreased dendritic spine and synaptic density starting from 9–13 weeks of age^9^. It would be interesting to examine whether these Tg mice show the observed aberrant splicing regulation at 2–3 months of age. Third, in ALS patient-specific induced-pluripotent stem cells, including ALS patient-derived VCP(R155C and R191Q), SOD1(A4V), and FUS(R521G) mutations, increased intron retention is a dominant feature during early neural differentiation^22,23^, which is in contrast to the most frequent exon skipping type detected in our study. Although we could not exclude that each ALS mutation has different effects on splicing regulation, it is also possible that aberrant splicing events in ALS are altered depending on neural differentiation stage and maturation. Finally, we were unable to confirm the elevated sema3G protein levels as well as protein-level changes resulting from aberrant splicing in patient samples. We aim to examine these levels in patient CSF in future studies. This is crucial as secreted sema3G is a potential biomarker for evaluating disease stage and its downstream factors, neuropilin-2/PlexinA4 receptor and Rac1, may also represent therapeutic targets in ALS/FTD treatment.

Nevertheless, our study identified extensive splicing changes mediated by mutant FUS in brain tissues during the early stages of ALS/FTD and confirmed the notion that dysfunction in RNA processing is a key aspect of neurodegeneration associated with ALS/FTD. Although further study is needed to validate whether aberrant splicing regulation leads to abnormal changes in protein levels and cellular functions linked to neurodegeneration, our study indicates that strategies targeting this aberrant splicing regulation may be a useful approach for the treatment of the motor/cognitive deficits of ALS/FTD.

## Materials and Methods

### Mice

Generating ΔNLS-FUS Tg mice has been described in detail in our previous study^9^; briefly, an NLS deleted mutant of human FUS cDNA was inserted into the pTSC21k vector containing the murine Thy1.2 expression cassette. The Tg mice were backcrossed for more than five generations with C57BL6/J mice. All mice used in this study were individually housed in ventilated micro-isolation cages under controlled temperature, humidity, lighting, and ventilation conditions with free access to pellet food and sterilized water during the experiments. DNA was isolated from ear clips and genotyping was performed using the following primer pair: 5′-AAGAAGACCTGGCCTCAAACG-3′ and 5′-TATCCCTGGGGAGTTGACTG-3′. Animal research protocols employed in this study were approved by the Committee on Animal Care and Use, Keio University (09217- [4]) and KAN Research Institute, Inc. and were conducted according to the Animal Experimentation Guidelines of Keio University School of Medicine and KAN Research Institute, Inc.

### Immunohistochemical staining

Mice were deeply anesthetized with the MMB combination anesthetic (i.e., 0.3 mg/kg of medetomidine hydrochloride, 4.0 mg/kg of midazolam, and 5.0 mg/kg of butorphanol tartrate) and then perfused intracardially with freshly prepared 2% paraformaldehyde in phosphate buffer for 10 min. Brains were dissected and soaked in the same fixative for 2 h on ice. For cryoprotection, the tissue pieces were placed into 20% sucrose solution for 4 h and 25% sucrose solution overnight, after which they were quickly frozen using liquid nitrogen and stored at −80 °C. Serial 7-µm-thick sections were cut using a cryostat (Leica Biosystems, Wetzlar, Germany). The sections were blocked with 1% BSA/10% normal donkey serum/0.5%Triton X-100/M.O.M. Blocking Reagent in PBS for 1 h at room temperature, followed by incubation with the primary anti-c-Myc 9E10 (1:100; sc-42; Santa Cruz Biotechnology, Dallas, TX) and FUS polyclonal (1:100; PA5-23696; Thermo Fischer Scientific, Waltham, MA) antibodies overnight. The sections were then incubated with the secondary antibodies Cy3 anti-mouse IgG (1:100; 715-166-151; Jackson ImmunoResearch Laboratories, West Grove, PA) and Alexa Fluor 488 anti-rabbit IgG (1:100; 711-546-152; Jackson ImmunoResearch Laboratories) for 1 h at room temperature. The embedded samples were imaged with a laser scanning confocal microscope (LSM700; Carl Zeiss, Oberkochen, Germany).

### RNA extraction

Total RNA was isolated from FCx, Hp, and lumbar SC tissues from 6-month-old mice [Wt mice, n = 4 (two males and two females); Tg mice, n = 4 (two males and two females)] using TRIzol reagent (15596–018; Invitrogen, Carlsbad, CA) according to manufacturer’s instructions. RNA samples were digested with the RNase-free DNase Set (79254; Qiagen, Hilden, Germany) to remove genomic DNA and further purified using the RNeasy Kit (74104; Qiagen). RNA sample quality was determined using the Agilent 2100 Bioanalyzer (Agilent Technologies, Santa Clara, CA) using RNA 6000 NanoChips (5067–1511; Agilent Technologies). RNA samples with an RNA integrity number (RIN) higher than 7 were used for further analysis, including qRT-PCR and RNA sequencing.

### mRNA-seq transcriptome analysis and identification of differential alternative splicing events

Total RNA was extracted from FCx, Hp, and SC tissue samples using TRIzol reagent according to manufacturer’s instructions, after which paired-end sequencing libraries were prepared by BGI JAPAN (Kobe, Japan) using TruSeq Stranded mRNA Sample Prep Kit (RS-122-2101; Illumina, San Diego, CA). Quality control, cluster generation, and sample sequencing were performed on the Illumina HiSeq 4000 platform (101 bp paired-end). Raw reads were quality checked using FastQC (v. 0.11.7), trimmed using Trimmomatic (v. 0.36), and mapped using HISAT2 (v. 2.1.0). For determination of the cell composition of tissue samples, marker genes for the specific cell types were chosen from the study published by Barham *et al*.^24^. DEGs were identified using DESeq2 (v. 3.4.1) and functional gene ontology analysis (padj < 0.05, |log2FC | ≧ 1) was performed using DAVID bioinformatics (https://david.ncifcrf.gov/) and KEGG (Kyoto Encyclopedia of Genes and Genomes)^25^. Differential alternative splicing genes were screened using rMATS (v. 3.1.0). Motif enrichment analysis of the RBPs (Supplementary dataset 15; RBP List for rMAPS) in the vicinity of the differentially regulated exons of exon skipping events in the Tg mice were analyzed using rMAPS server 17 (http://rmaps.cecsresearch.org/)^26^ and all skipped/retained exon events identified by MATS were used as the input. Significance level was determined by comparison to a “background set” of 32,114 unaffected alternative exons (rMATs FDR > 50%) of genes that showed expression in these tissues (FPKM > 5.0). Only events showing P ≤ 0.05, FDR ≤ 0.05, and a minimum inclusion level difference ≥0.1 were considered.

### qRT-PCR

cDNA was synthesized from 1 μg of the abovementioned total RNA using Superscript III First-Strand Synthesis System (12574030; Thermo Fisher Scientific). Expression levels of *Sema3g, Hbb-b2, Hba-a2*, and *Alas2* were quantified using the DyNAmo ColorFlash SYBR Green qPCR Kit (F-416L; Thermo Fisher Scientific) on a PikoReal Real-Time PCR System (12675885; Thermo Fisher Scientific). Real time PCR was carried out with the following cycling conditions: initial denaturation at 94 °C for 3 minutes, 45 cycles of 94 °C for 15 seconds, 60 °C for 1 minute, followed by 95 °C for 15 seconds, and 60 °C for 15 seconds. The primer sets are listed in Supplementary dataset 16 (qRt-PCR primers).

### Statistical analysis

Student’s *t*-test (Fig. 1) and Tukey-Kramer test (Supplemental Fig. S3) were used to analyze the data. Statistical analyses were performed using StatView (v. 5.0; StatView, Berkeley, CA) or SPSS Statistics (v. 24; IBM, Armonk, NY).

### Ethical approval

Animal research protocols used in this study were approved by the Committee on Animal Care and Use, Keio University (09217- [4]) and KAN Research Institute, Inc., and conducted according to the Animal Experimentation Guidelines of Keio University School of Medicine and KAN Research Institute, Inc.

## Supplementary information

Supplementary Information.

Supplementary Dataset 1.

Supplementary Dataset 2.

Supplementary Dataset 3.

Supplementary Dataset 4.

Supplementary Dataset 5.

Supplementary Dataset 6.

Supplementary Dataset 7.

Supplementary Dataset 8.

Supplementary Dataset 9.

Supplementary Dataset 10.

Supplementary Dataset 11.

Supplementary Dataset 12.

Supplementary Dataset 13.

Supplementary Dataset 14.

Supplementary Dataset 15.

Supplementary Dataset 16.

## Data Availability

Supporting data and protocols are made available without restrictions.
