# Supplementary material for: Extensive splicing changes in an ALS/FTD transgenic mouse model overexpressing cytoplasmic fused in sarcoma
Source: Sci Rep. 2020 Mar 17;10:4857. doi: 10.1038/s41598-020-61676-x (PMC7078223; doi:10.1038/s41598-020-61676-x)
Supplement: Supplementary file 1 — Supplementary Information. [file 41598_2020_61676_MOESM1_ESM.pdf]

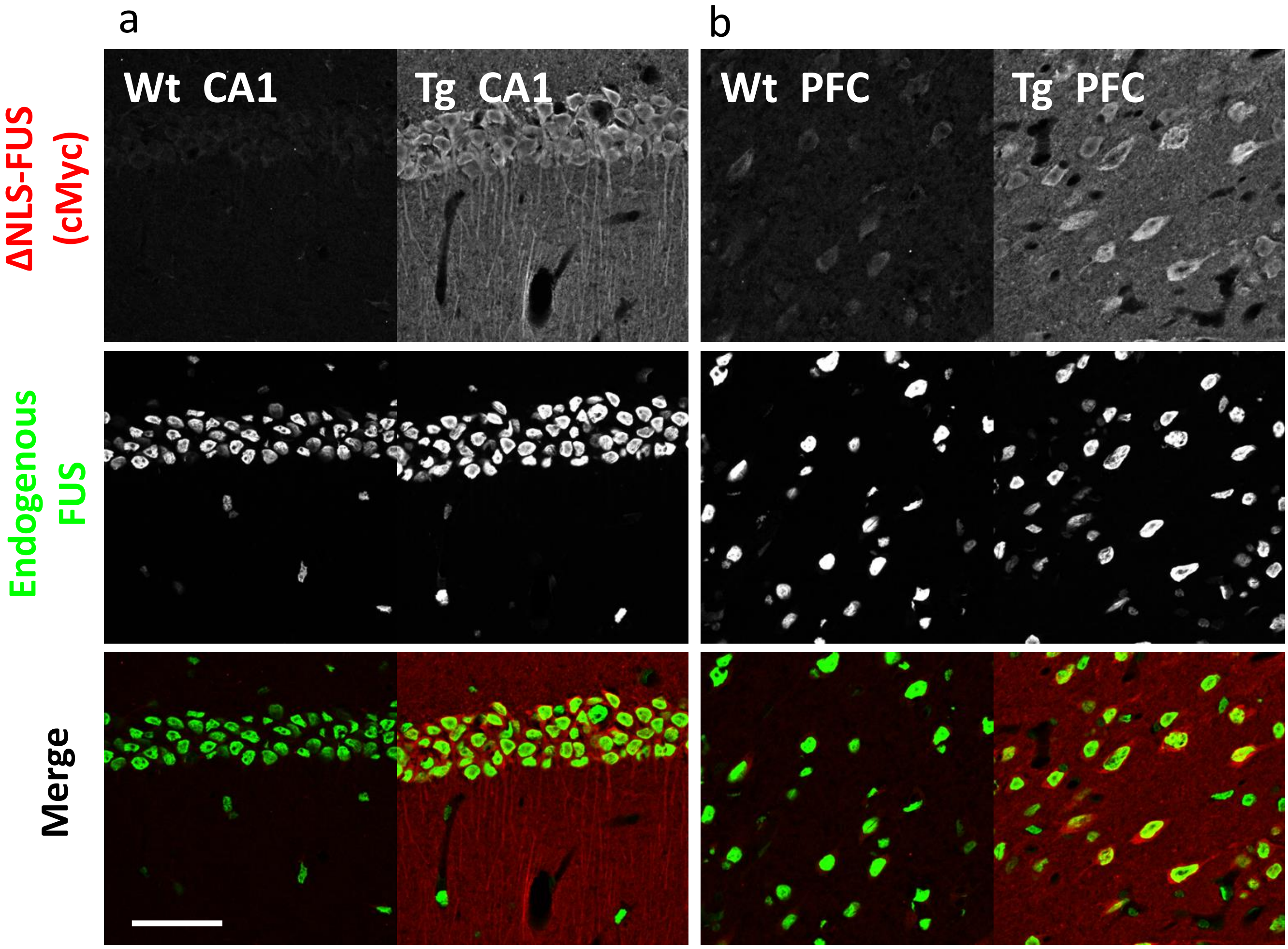

Supplementary Figure S1. Representative immunohistochemistry depicting the subcellular localization of endogenous FUS and exogenous mutant FUS (ΔNLS-FUS) in the brain tissues of 6-month-old wild-type (Wt) and transgenic (Tg) mice. (a) CA1. (b) Prefrontal cortex (PFC). Red represents ΔNLS-FUS staining and green represents endogenous FUS staining; scale bar: 50 μm.

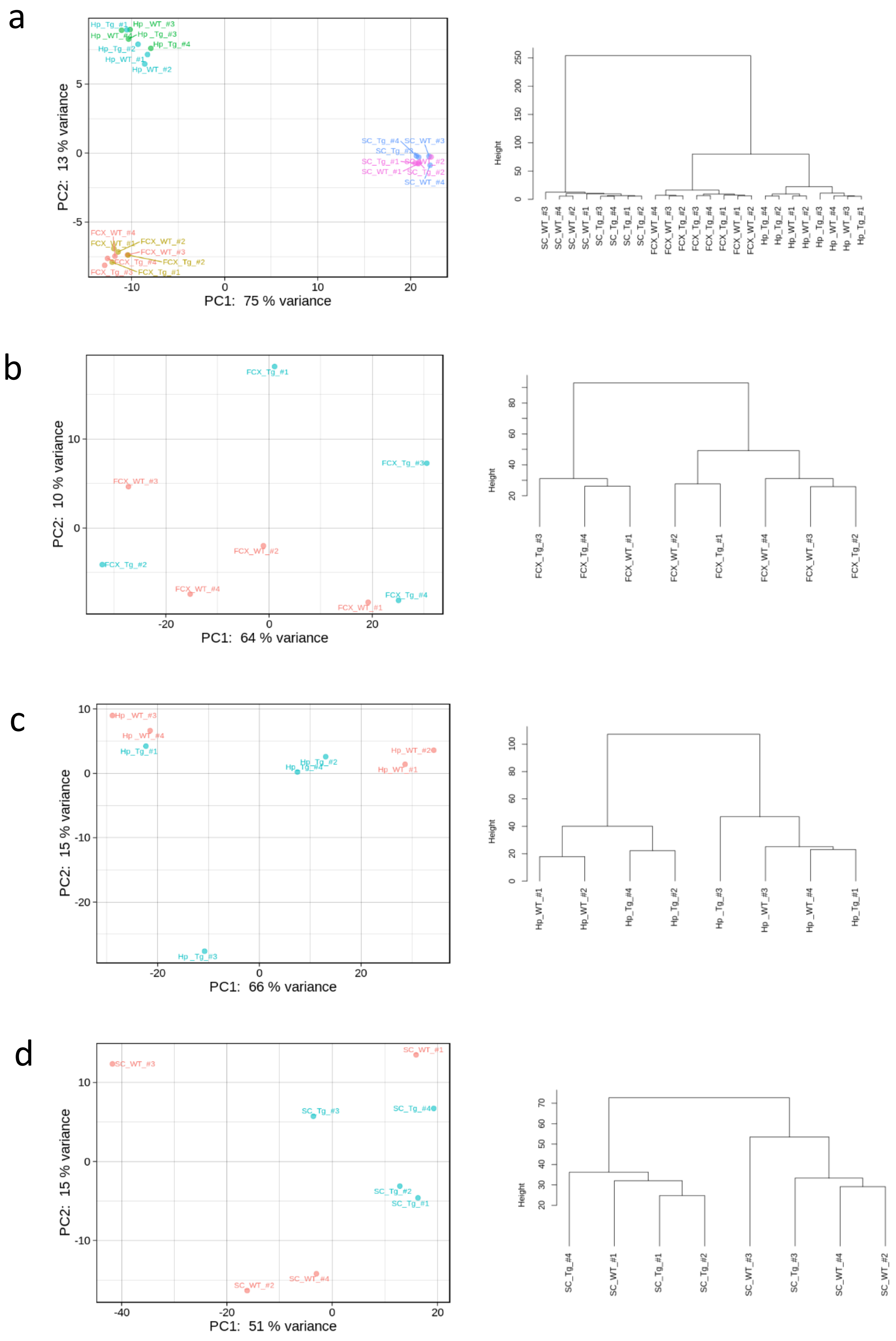

Supplemental Figure S2. RNA sequence analysis of three tissues samples from Wt and Tg mice (each n = 4). Hierarchical clustering (left) and PCA plot (right) of (a) all tissues, (b) FCx, (c) HP, (d) and SC. The tissues do not show separation between Wt and Tg mice. FCx, frontal cortex; Hp, hippocampus; SC, spinal cord.

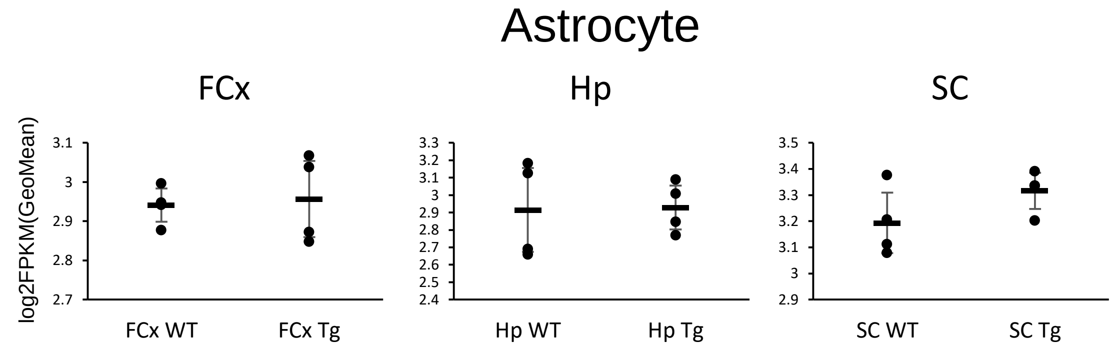

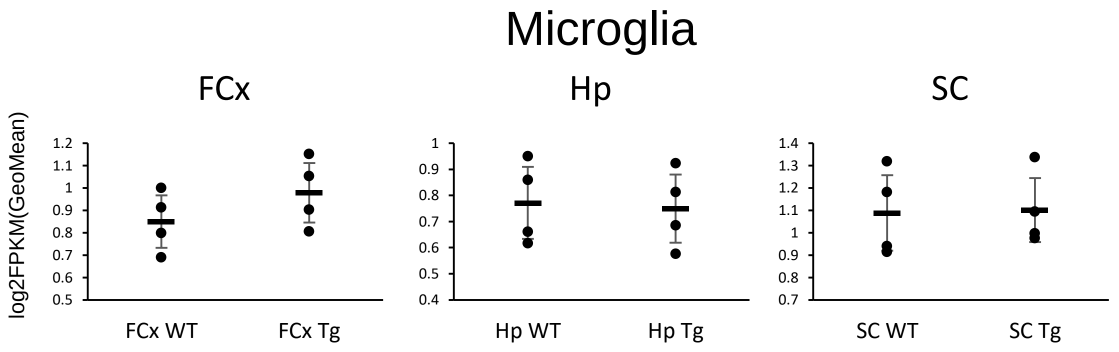

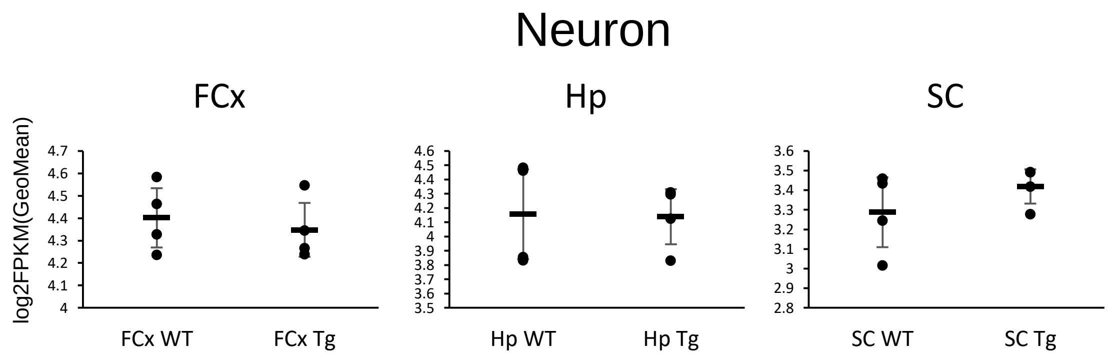

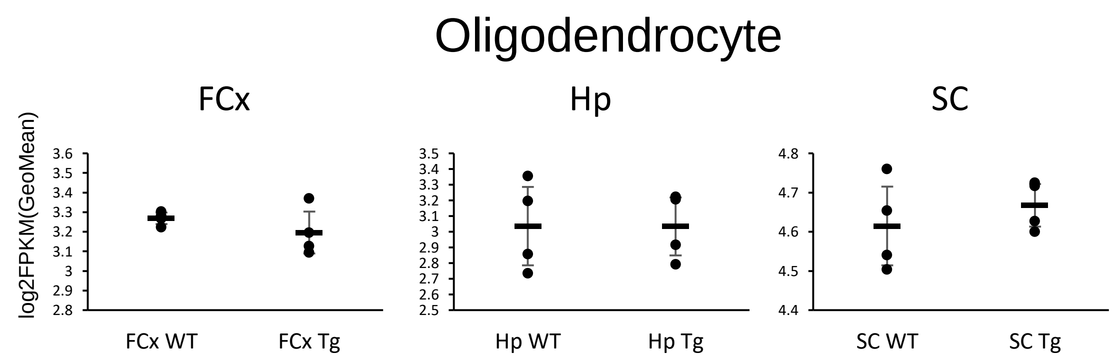

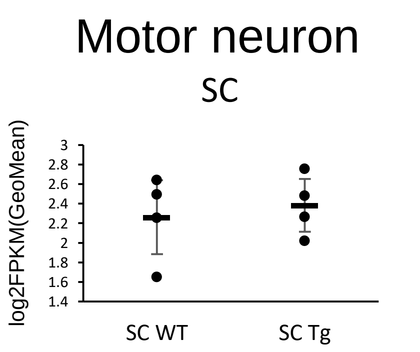

Supplemental Figure S3. Cell-type composition of FCx, Hp, and SC samples. Changes in specific cell types indicated by the log2FPKM(GeoMean) values of cell-type marker genes. Statistical differences in cell-type composition were calculated by the Tukey-Kramer test. Data do not show significant differences.

Extensive splicing changes in an ALS/FTD transgenic mouse model overexpressing cytoplasmic fused in sarcoma.
Daisuke Ito, Ryota Taguchi, Maki Deguchi, Hideaki Ogasawara, Eiji Inoue

Supplementary Table S1. List of differentially expressed genes (padj < 0.05, |log2FC| ≧ 1) in SC tissue of ∆NLF-FUS Tg mice

Tissue	Gene_name	log2 Fold Change	adjusted p-value	Wt. ave. ±S.D.（FPKM）	Tg ave. ±S.D.（FPKM）	Description	KEGG Pathway
SC	Hbb-b2	-1.64	1.50E-07	97.73±30.66	5.35±0.83	hemoglobin, beta adult t chain	
	Hba-a2	-1.46	5.34E-07	232.27±59.31	88.94±28.86	hemoglobin alpha, adult chain 1	
	Gm16867	-1.02	5.34E-06	17.92±7.77	97.73±30.66		
	Mpo	-2.65	1.23E-05	2.18±0.78	232.27±59.31	myeloperoxidase	Phagosome // Transcriptional misregulation in cancer
	Alas2	-1.59	4.59E-05	3.94±1.04	17.92±7.77	aminolevulinic acid synthase 2, erythroid	Glycine, serine and threonine metabolism // Porphyrin and chlorophyll metabolism // Metabolic pathways
	Hba-a1	-1.37	7.46E-05	325.15±87.64	2.18±0.78	hemoglobin alpha, adult chain 1	African trypanosomiasis // Malaria
	Hbb-b1	-1.34	1.17E-04	166.48±54.81	3.94±1.04	hemoglobin, beta adult t chain	
	Slc4a1	-1.61	0.02	1.34±0.70	325.15±87.64	solute carrier family 4 (anion exchanger), member 1	Collecting duct acid secretion

Extensive splicing changes in an ALS/FTD transgenic mouse model overexpressing cytoplasmic fused in sarcoma.
Daisuke Ito, Ryota Taguchi, Maki Deguchi, Hideaki Ogasawara, Eiji Inoue

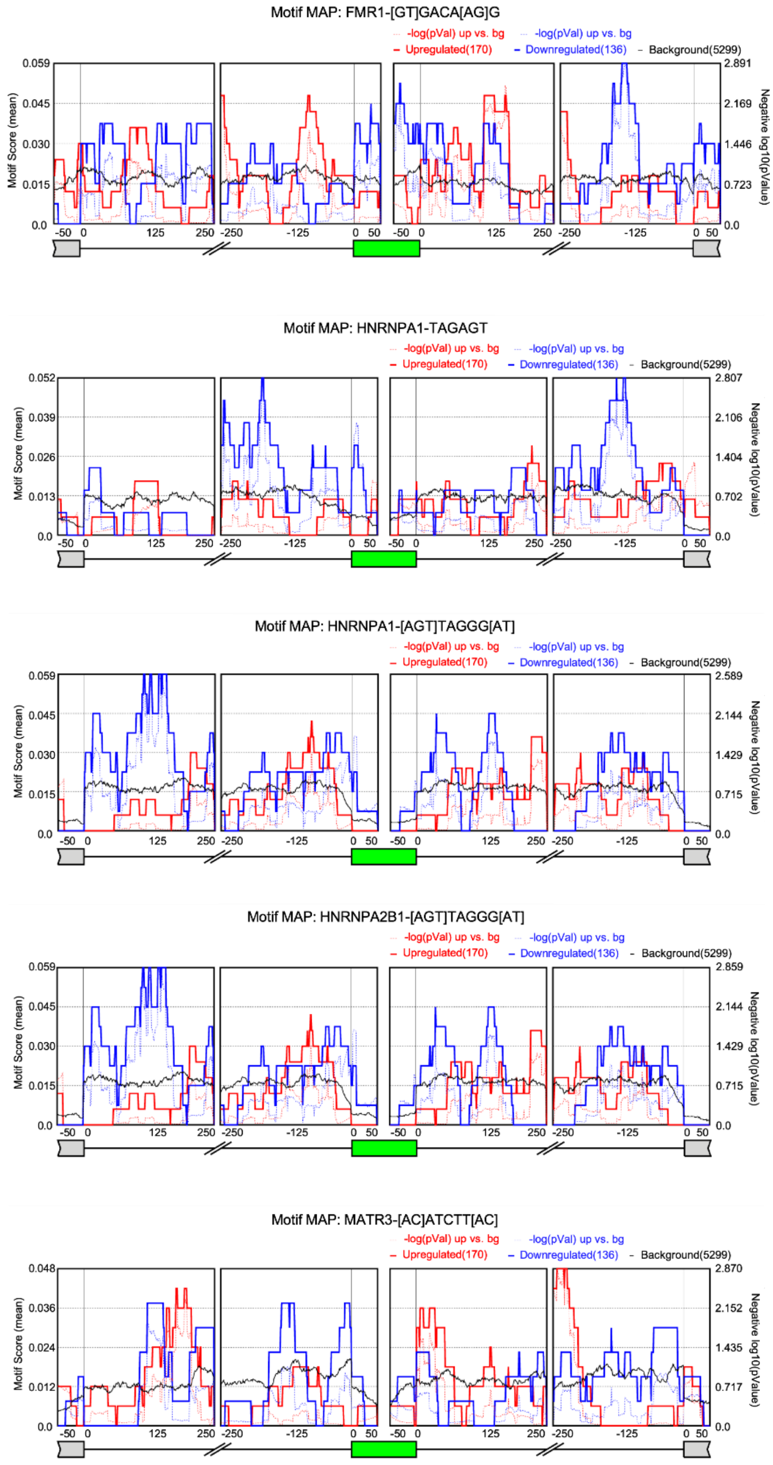

Supplemental Figure S4. Maps of RNA-binding protein motifs showing enrichment up- and downstream exons with enhanced (red) or silenced (blue) skipping in FCx tissues of Tg mice.

Extensive splicing changes in an ALS/FTD transgenic mouse model overexpressing cytoplasmic fused in sarcoma.
Daisuke Ito, Ryota Taguchi, Maki Deguchi, Hideaki Ogasawara, Eiji Inoue

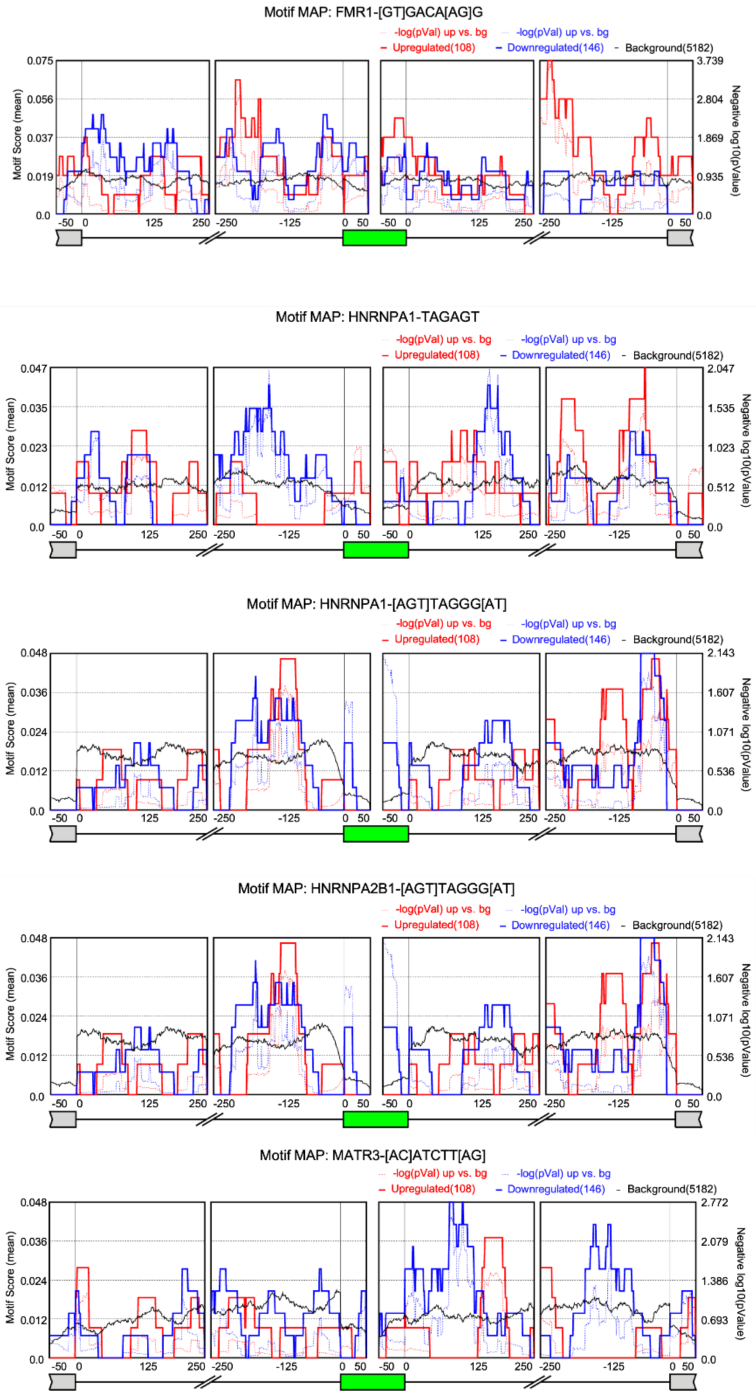

Supplemental Figure S5. Maps of RNA-binding protein motifs showing enrichment up- and downstream exons with enhanced (red) or silenced (blue) skipping in Hp tissues of Tg mice.

Extensive splicing changes in an ALS/FTD transgenic mouse model overexpressing cytoplasmic fused in sarcoma.
Daisuke Ito, Ryota Taguchi, Maki Deguchi, Hideaki Ogasawara, Eiji Inoue

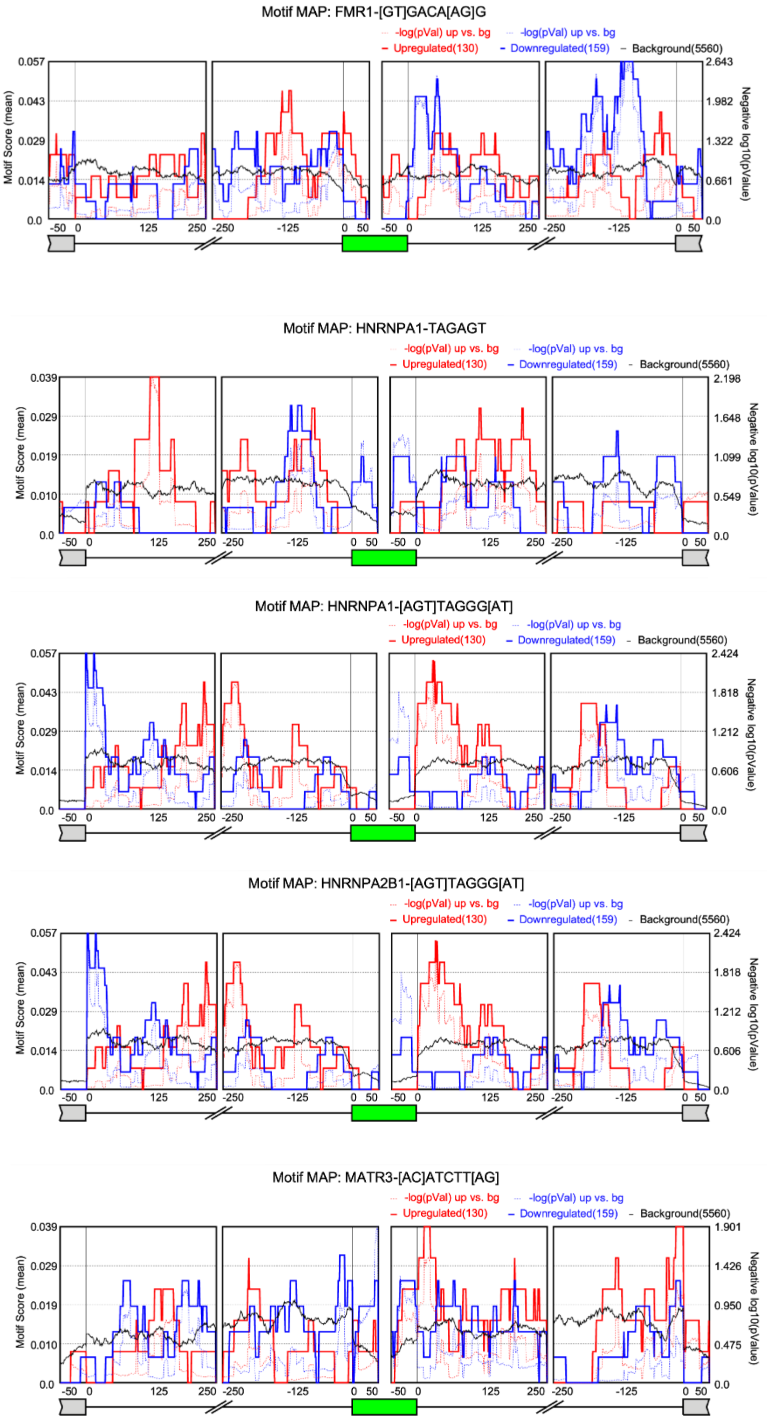

Supplemental Figure S6. Maps of RNA-binding protein motifs showing enrichment up- and downstream exons with enhanced (red) or silenced (blue) skipping in SC tissues of Tg mice.
